# Small Molecule Pytren-4QMn Metal Complex Slows down Huntington’s Disease Progression in Male zQ175 Transgenic Mice

**DOI:** 10.3390/ijms242015153

**Published:** 2023-10-13

**Authors:** Marián Merino, Sonia González, Mª Carmen Tronch, Ana Virginia Sánchez-Sánchez, Mª Paz Clares, Antonio García-España, Enrique García-España, José L. Mullor

**Affiliations:** 1 Bionos Biotech SL, Biopolo Hospital La Fe, 46026 Valencia, Spain; mmerino@bionos.es (M.M.); sgonzalez@bionos.es (S.G.); tronchma@alumni.uv.es (M.C.T.); sanchezav78@gmail.com (A.V.S.-S.); antoniogem85@gmail.com (A.G.-E.); 2 Departamento de Química Orgánica e Inorgánica, Instituto de Ciencia Molecular, Universidad de Valencia, 46980 Valencia, Spain; m.paz.clares@uv.es (M.P.C.); enrique.garcia-es@uv.es (E.G.-E.)

**Keywords:** Mn (II) complexes, 4QMn, Huntington’s disease, Q175 mice, polyglutamine diseases, sex differences

## Abstract

Huntington’s disease (HD) is an inherited neurodegenerative disorder considered a rare disease with a prevalence of 5.7 per 100,000 people. It is caused by an autosomal dominant mutation consisting of expansions of trinucleotide repeats that translate into poly-glutamine enlarged mutant huntingtin proteins (mHTT), which are particularly deleterious in brain tissues. Since there is no cure for this progressive fatal disease, searches for new therapeutic approaches are much needed. The small molecule pytren-4QMn (4QMn), a highly water-soluble mimic of the enzyme superoxide dismutase, has shown in vivo beneficial anti-inflammatory activity in mice and was able to remove mHTT deposits in a C. elegans model of HD. In this study, we assessed 4QMn therapeutic potential in zQ175 neo-deleted knock-in mice, a model of HD that closely mimics the heterozygosity, genetic injury, and progressive nature of the human disease. We provide evidence that 4QMn has good acute and chronic tolerability, and can cross the blood–brain barrier, and in male, but not female, zQ175 mice moderately ameliorate HD-altered gene expression, mHtt aggregation, and HD disease phenotype. Our data highlight the importance of considering sex-specific differences when testing new therapies using animal models and postulate 4QMn as a potential novel type of small water-soluble metal complex that could be worth further investigating for its therapeutic potential in HD, as well as in other polyglutamine diseases.

## 1. Introduction

Huntington’s disease (HD) is a rare inherited neurodegenerative disorder with a prevalence rate of 5.7/100,000 in populations of European ancestry [1,2]. It is caused by an autosomal dominant mutation in the first exon of the huntingtin (*HTT*) gene, resulting in a more than fifty-fold increase in the number of *HTT* constitutive CAG trinucleotide repeats (CAG repeats) and the consequent translation of poly-glutamine (poly-Q) enlarged huntingtin proteins [3]. Although spliced mHTT containing the mutant first exon are ubiquitously expressed, they exhibit high toxicity in brain tissues, particularly in the striatum and cortex [4,5].

Currently, there is no known cure for HD, and the development of novel therapeutic approaches to improve the quality of life of HD patients is highly needed [6,7]. Most therapeutic approaches aim to reduce the presence of mHTT in the brain, as it is the causative agent of the disease and its most harmful effects occur in brain tissues, particularly in the striatum and cortex [1,5,7,8]. Reduction of mHTT in the brain has been achieved using genetic strategies like antisense oligonucleotides (ASOs), siRNAs or miRNAs, which have shown significant improvements in HD phenotype in different mouse models [6,7]. Although these therapies are being tested in clinical trials, they are difficult to apply and require specialized personnel and techniques, such as surgical delivery of a viral vector for RNAi, or lumbar puncture for ASO [7,9]. Recently, we reported that pytren-4QMn (4QMn) enhances ubiquitin–proteasome and autophagy activity to degrade mHTT in human cultured cells and worm (*C. elegans*) models of HD [10]. The small molecule 4QMn is a highly water-soluble (>0.4 g/mL) mimic of the enzyme superoxide dismutase [11] and displays proven antioxidant activity in prokaryotes (bacteria), fungi (yeast), and animals (medaka fish and mice) [12,13]. Additionally, antioxidant compounds like vitamin E, Coenzyme Q, and creatine have demonstrated beneficial effects in transgenic mouse models of HD [14,15]. Moreover, systemic administration is important because mHTT expression affects non-neuronal tissues as well, causing impairments in many organs and tissues, including the heart, lungs, bones, and skin [16,17,18].

In view of the above information, we hypothesized that 4QMn could also be beneficial in polyglutamine neurodegenerative diseases such as HD. To evaluate the therapeutic potential of 4QMn, we utilized zQ175 neo-deleted knock-in (zQ175neo) mice, a mouse model of HD that closely mimics the heterozygosity, genetic injury, and progressive nature of the human disease [19,20]. These transgenic mice, which carry the human HD mutation inserted into the mouse gene, form mHtt aggregates in various tissues, including the striatum, and show a slow-progressing development of motor alterations such as hind leg mobility, orientation, strength, and equilibrium that, like in humans, precedes neuropathology, and becomes intense and generalized with age [19,20].

Preclinical studies in animal models of disease are crucial in drug development processes to assess the toxicity and in vivo efficacy of new compounds. In this preliminary pilot study, we demonstrate that 4QMn exhibits good acute and chronic tolerability and can cross the blood–brain barrier, and its sustained oral administration partially reverts some biochemical HD-related characteristics and attenuates the progression of the behavioral phenotype in male, but not female, zQ175neo mice.

## 2. Results

### 2.1. Assessment of 4QMn Acute and Chronic Tolerability

To evaluate the acute and chronic toxicity of 4QMn, we used C57BL/6J mice, which are the background strain of the zQ175neo transgenic mice. In the case of IP delivery, a first dose of 10 mg/kg was tested for one mouse, and it died 1 h later. Then, we decided to use a dose 10 times lower (1 mg/kg) in 6 mice and no adverse effects were observed. Therefore, maximum tolerated doses were established at 1 mg/kg for intraperitoneal (IP) delivery and 500 mg/kg for intragastric (IG) infusion (see Supplementary Table S1). For the acute toxicity assessment, a single IP injection of either NaCl 0.9% or 4QMn was administered at doses of 0.1 and 1 mg/kg to five mice and monitored for 14 days. No significant differences were found between the treated and control mice in terms of body weight or plasma levels of alanine aminotransferase (ALT), a liver damage marker (see Supplementary Table S2 and Figure S1). For the chronic toxicity evaluation, 5 mice were injected IP with 1 mg/kg of 4QMn 3 days a week for 28 days, or 3 mice were administered IG 0, 5, 10 or 50 mg/kg of 4QMn every 3 days for 90 days. In both cases, no significant differences were found in body weight or plasma levels of ALT. Additionally, no observable gross lesions or histopathological alterations were found in the liver, except in the highest IG dose (50 mg/kg) group, in which only one mouse survived and showed evident hepatic inflammation (see Supplementary Table S3 and Figure S2).

### 2.2. 4QMn Can Cross the Blood–Brain Barrier

**Figure 1:**
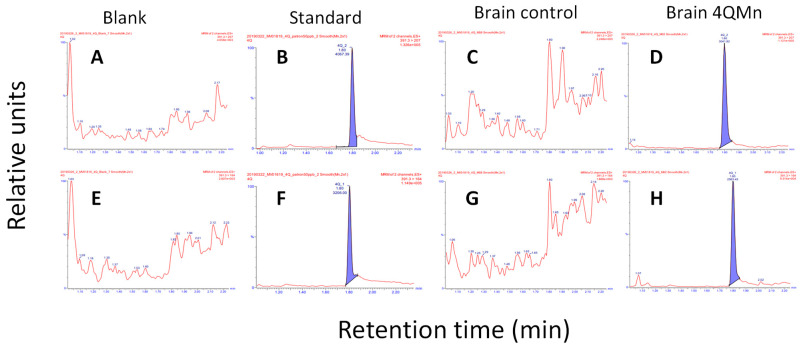
Detection of 4QMn in mouse brain. LC–MS–MS representative chromatograms of pytren-4Q at two mass charge transitions: 391.3 > 207 (**A**–**D**) and 391.3 > 164 (**E**–**H**). (**A**,**E**) Blank. (**B**,**F**) Standard 50 ng/mL. (**C**,**G**) Brain control. (**D**,**H**) Brain 4QMn (5 mg/kg, IN). Filled in blue pytren-4Q specific chromatographic peak at 1.8 min (retention time).

Due to the highly selective blood–brain barrier, which isolates brain tissues from the rest of the body, and the significance of brain tissues in the development of HD pathology, it was essential to investigate whether 4QMn could penetrate the brain tissue. To accomplish this, an assay based on liquid chromatography coupled with tandem mass spectrometry (LC-MS/MS) was developed. In this assay, pytren-4Q generated a highly specific and selective signal consisting of a chromatographic peak at 1.8 min (retention time) and two mass charge transitions of 391.3 > 207 and 391.3 > 164 (Figure 1). Results indicated that following an intranasal delivery of 5 mg/kg of 4QMn or an IG infusion of 50 mg/kg of 4QMn, the compound 4QMn was detected in brain tissues (Figure 1 and Supplementary Table S4). However, although after an IG infusion of 20 mg/kg, we detected pytren-4Q in liver tissue and in blood serum, the levels in brain tissue were below the limit of detection of the assay (Supplementary Figure S3 and Table S5).

### 2.3. 4QMn Treatment Ameliorates the Progression of the HD Phenotype in Males

**Figure 2:**
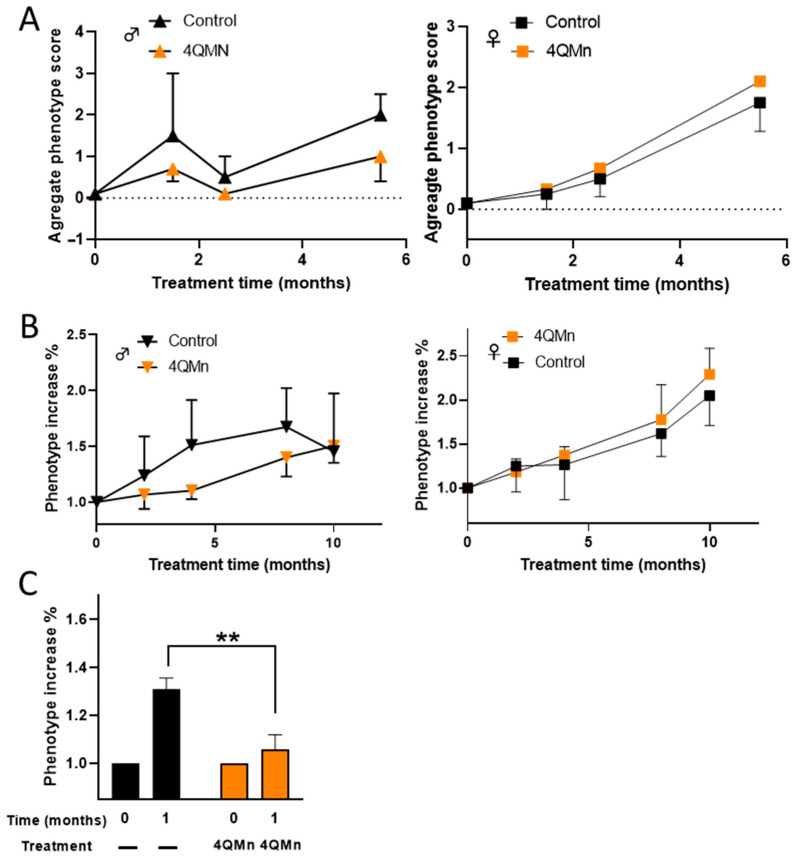
4QMn slows down HD disease phenotype in zQ175neo males. (**A**) NaCl 0.9M or 20 mg/Kg 4QMn was administered IG for 5.5 months to 3 months old male (triangles) and female mice (squares). Values are the mean ± SEM of the combined scores of the motor tests. (**B**) Ad libitum 4QMn treatment was administered at 1.2 mM in drinking water 4.5 days a week for 10 months to 12 months old male and female mice. Values represent the mean ± SEM of the increase in the disease phenotype relative to the phenotype at the start of the treatments. Time 0 absolute values were 4.20 ± 0.66 (n = 5) and 4.66 ± 0.49 (n = 6) for control and treated males, and 3.66 ± 0.92 (n = 6) and 3.33 ± 0.33 (n = 6) for control and treated females, respectively. (**C**) Ad libitum 4QMn treatment was administered at 1.2 mM in drinking water 7 days a week for one month to 15-month-old male mice. Black bars represent non-treated mice (control) and orange bars represent mice treated with 4QMn. Values are the mean ± SEM of the increase in disease phenotype relative to the phenotype at time 0. Time 0 absolute values were 6.40 ± 0.41 (n = 15) for control mice and 6.73 ± 0.39 (n = 15) for 4QMn treated mice. ** *p* = 0.0030.

In order to evaluate the therapeutic potential of 4QMn, heterozygous zQ175neo mice were used and we monitored the progression of the HD phenotype using four different motor tests (equilibrium, hind leg mobility, orientation, and kyphosis—abnormal curvature of spines). The combined scores of these tests ranged from 0 to 12 (see Section 4) [21]. In a first pilot study, 19 mice (5 males and 14 females) that were approximately 3 months old were used. These mice were treated every 3 days, via IG infusion with NaCl 0.9% or 20 mg/kg of 4QMn, for 5.5 months. Although the HD motor phenotype was not present at the start of the study (score 0), it only reached 25% of the maximum score at the end of treatment. However, the small number of mice and the lack of HD phenotype development precluded a proper evaluation. Nevertheless, when male and female mice were analyzed separately, it was observed that 4QMn treatment seemed to impede the progression of the HD phenotype only in males (Figure 2A). In order to allow the HD phenotype to develop, an efficacy study was conducted over a period of 10 months with 27 older mice (approximately 12 months old at the start of the study). To avoid affecting the mice’s well-being as is the case with IP or IG administrations over long periods, 4QMn was administered in the drinking water 4.5 days a week at 1.2 mM, equivalent to 175 mg/kg/day. Throughout the study, 3 control mice (2 females and 1 male) and 1 treated male died. No decrease in body weight equal to or greater than 20% was observed in any group of mice, although males showed an overall lower body weight than females (Supplementary Figure S4). In this preliminary efficacy study, although without statistical significance, 4QMn treatment seemed to ameliorate the progression of the HD phenotype only in males (Figure 2B). Based on these results, a second efficacy study was conducted with 33 males who were 15 months old, and the treatment (4QMn 1.2 mM) was administered in drinking water seven days a week for one month. In this case, results showed that the treatment significantly (*p* = 0.0030) hampered the progression of the HD phenotype in the treated group (Figure 2C). 

### 2.4. 4QMn Partially Recovered Darpp32 Expression and Reduced the Number of Nuclei with mHTT Granules in Male Striatum

**Figure 3:**
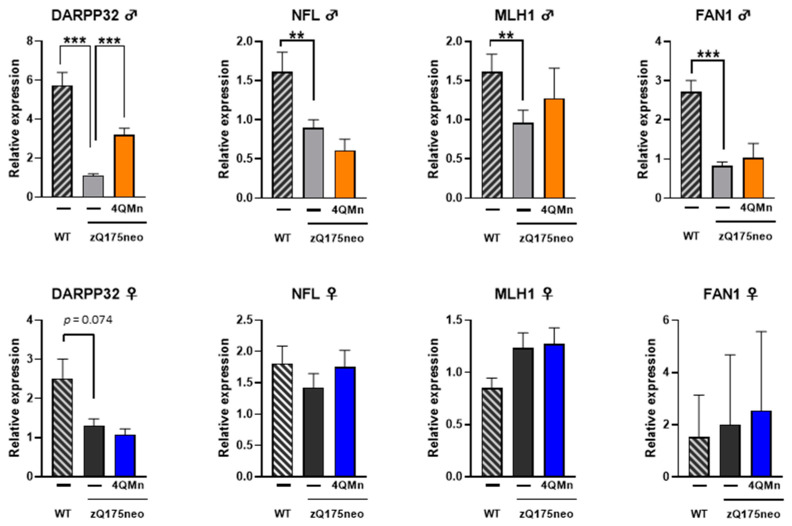
4QMn treatment reverts HD downregulation of *Darpp32* in male zQ175neo mice. Effect of 4QMn chronic treatment for ten months in RNA expression of the indicated genes in the striatum. The graphs show mRNA expression measured by real-time PCR of 4QMn treated (orange bars for male and blue bars for female) and control (light grey bars for male and dark grey bars for female) zQ175neo and age-matched WT mice (striped bars). Expression values are given in percentage relative to untreated zQ175neo mice. Upper panel male and lower panel female mice. Note that 4QMn treatment only reverted *Darpp32* in male mice. *p* values *** *p* < 0.001, ** *p* < 0.01.

HD pathogenesis has been associated with transcriptional dysregulation in the striatum, which is one of the earliest and most severely affected brain regions [4,5]. Some genes that impact CAG instability, such as Fanconi Anemia Associated Nuclease 1 (*Fan1*) and MutL Protein Homolog 1 (*Mlh1*), as well as Neurofilament Light Chain (*Nfl*), a marker of disease severity, and cyclic-AMP-regulated phosphoprotein (*Darpp32*), which is inversely correlated with mHtt accumulation, have been found to be dysregulated in HD [1,22,23]. In this study, it was observed that *Darpp32* expression was significantly downregulated in both male and female zQ175neo mice compared to age-matched WT mice (Figure 3). However, *Nfl*, *Mlh1,* and *Fan1* were only downregulated in male zQ175neo mice, and there were no differences in female gene expression between WT and zQ175neo mice (Figure 3). Treatment with 4QMn for ten months significantly reverted the downregulation of *Darpp32* only in males, but it did not significantly affect other parameters in either male or female mice (Figure 3).

**Figure 4:**
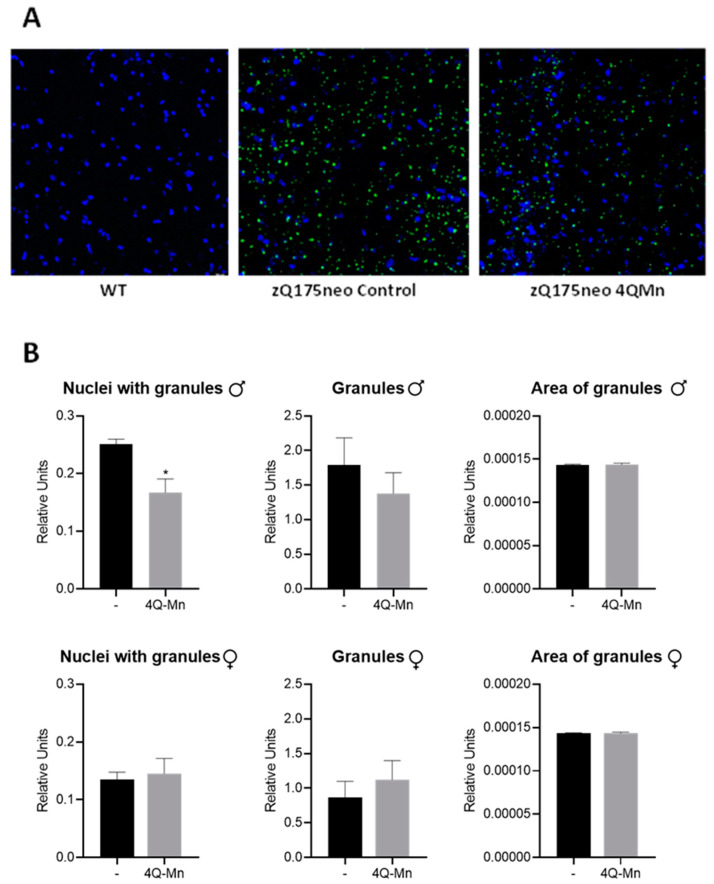
4QMn decreases the number of nuclei with mHtt aggregates in striatum of male mice. Effect of 4QMn treatment in the number and area of granules, and number of nuclei with granules in striatum. (**A**) Representative images of stained nuclei (blue) and immunodetected mHtt aggregates (green) in WT and treated and control zQ175neo mice. Magnification 20×. (**B**) Relative quantification by immunohistochemistry of the number of nuclei with granules, number of granules, and area of granules in 4QMn treated and control male (upper panel) and female (lower panel) zQ175neo mice. * *p* < 0.05.

On the other hand, immunohistochemistry was used to determine the effects of 4QMn treatment on the accumulation of mHtt in granules in the striatum, which is a hallmark of HD. The analysis showed that treatment with 4QMn decreased the number of nuclei with granules in male zQ175neo mice in comparison with control mice. However, no significant effects were observed in other parameters in either male or female zQ175neo mice (Figure 4).

## 3. Discussion

In the present study, we have evaluated the therapeutic potential of the small antioxidant molecule 4QMn in improving HD phenotype in male and female zQ175neo mice. We found that 4QMn has good tolerability, can cross the blood–brain barrier, and ameliorates biochemical HD biomarkers and the progression of the HD phenotype in male, but not in female, zQ175neo mice. Previous studies have shown that 4QMn has anti-inflammatory activity in mice and the potential to degrade mHTT by inducing autophagy and activating the proteosome independently of its antioxidant activity in *C. elegans* and cell models of HD [10,13].

Huntington’s disease is a devastating neurodegenerative disorder for which there is currently no cure [24,25]. Like many other neurodegenerative disorders, HD displays impaired mitochondrial function and reactive oxygen species (ROS)-mediated damage, suggesting a close involvement of ROS in the pathology of HD [24]. However, the causal links between HD and antioxidants remain unclear [26,27]. In this context, antioxidant therapies in poly-Q diseases have failed in clinical trials, possibly due to the intricate and interconnected network relationships between oxidative stress, inflammation, and protein homeostasis (proteostasis) [26,27,28,29,30]. Nevertheless, in addition to genetic therapeutic approaches, the use of antioxidants or compounds targeting protein degradation machinery, such as proteasome activators and autophagy inducers, have shown multiple benefits in animal models of HD, and their pharmacological manipulation may potentially alleviate the disease progression in the future [27]. Thus, the development of novel therapeutic approaches, including combination therapies, is crucial for the treatment of this fatal condition [4].

4QMn has the potential to act as a multitasking molecule by reducing reactive ROS levels and enhancing mHTT degradation [10,13]. Moreover, 4QMn has metal scavenging properties and exhibits a high affinity for redox-active metals such as Cu and Fe, which tend to accumulate in neurons and glia in HD, suggesting that the prevention of their accumulation may be beneficial [11,12,26]. It should be noted that even if Mn in 4QMn is replaced by other metals like Cu and Fe within cells, the resulting molecule will retain its antioxidant capabilities (E. Garcia-España, personal communication). In addition, 4QMn has previously demonstrated anti-inflammatory/antioxidant activity in vivo in mice and medaka fish, and mHTT degradation activity in *C. elegans* models of HD [10,12,13]. Moreover, given its high water solubility, 4QMn can be easily administered orally through drinking water.

However, it should be noted that the effectiveness of the 4QMn treatment was limited to male Q175neo mice. A literature survey suggests that it can be attributed to differential Erk1/2 MAP kinase signaling between male and female Q175 mice. In this sense, in male, but not female, zQ175 mice, an agonist of metabotropic glutamate receptor 2 and 3 (mGluR2/3) resulted in a reduction of mHtt that correlated with the activation of autophagy in the striatum [31]. Furthermore, extracellular signal-regulated MAP kinase ERK1/2 phosphorylation was decreased in males but increased in females zQ175 mice after agonist treatment [31]. A reduction in ERK1/2 phosphorylation in the striatum was also observed in Q175 males treated with metformin, an inducer of autophagy that alleviates the progression of the HD phenotype [32]. Notably, 4QMn significantly reduced the oxidative stress-dependent phosphorylation of MAP kinase enzymes ERK1/2, p38, and JNK in cells challenged by an endotoxin stimulus [13]. Conversely, inhibition of ERK1/2 phosphorylation activates tuberous sclerosis complexes 1 and 2 (TSC1/2), which in turn induce autophagy downstream of the AMP-activated protein kinase (AMPK) enzyme [33,34]. Moreover, inhibition of the p38 MAPK pathway reduced the accumulation of aggregated and soluble forms of mHtt in cultured mouse striatal cells [35]. Currently, a p38α MAPK inhibitor, Neflamapimod, is being evaluated in early-stage HD patients [9]. Taken together, these observations suggest that the male-only efficacy of 4QMn treatment may be attributed, in part, to differential Erk1/2 MAP kinase signaling between male and female Q175 mice. The effectiveness of 4QMn treatment only in male Q175neo mice highlights the importance of considering sex-specific differences when testing new therapies using animal models of HD [31,36,37].

In conclusion, our findings provide compelling evidence that 4QMn is well-tolerated *in vivo*, can effectively cross the blood–brain barrier, and moderately ameliorates HD biomarkers such as *Darrp32* expression and mHtt aggregation in male Q175neo mice, as well as the behavioral phenotype of HD in middle and late stages of the disease after sustained administration through drinking water. In this sense, it will be interesting to investigate the chronic oral administration of 4QMn in early HD, before the onset of the disease symptoms. While further research is necessary to determine the full therapeutical potential and sex-specific effects of 4QMn, our results highlight the importance of considering sex-specific differences when testing new therapies using animal models of HD [38,39,40,41]. Nevertheless, 4QMn represents a promising novel small water-soluble metal-complex molecule with potential therapeutic value in HD and other poly-Q diseases, given its modest yet significant beneficial effects.

## 4. Materials and Methods

### 4.1. Animals

All animal experimental protocols were approved by the Animal Testing Ethics Committee (CEEA) of the Health Research Institute Hospital La Fe (IIS La Fe; Valencia, Spain) and were in accordance with the EU Directive 2010/63/EU on the protection of animals used for scientific purposes. These protocols include efforts to minimize animal suffering and the number of mice used. Animals were group caged and housed under a constant 12-h light/dark cycle and food and water were given ad libitum. Heterozygous zQ175neo HD mice carrying 180–220 CAG repeats were obtained from the Jackson Laboratory (B6J.129S1-Htttm1.1Mfc/190ChdiJ) and bred with background strain C57BL/6J to establish littermate-controlled male and female wild type (Wt) and heterozygous zQ175neo mice. We assessed zQ175neo mice HD motor phenotype using four tests: equilibrium, hind leg mobility, orientation, and kyphosis, as described in Guyenet et al., 2010. Each test was evaluated individually from 0 to 3, with 0 being the score of phenotypes with no apparent damage and 3 being the highest degree of severity. During some of the studies at different time intervals, 200 µL blood was obtained from the facial vein and stored at −80 °C to quantify alanine aminotransferase (ALT) activity. After the different studies, mice were sacrificed by cervical dislocation, and tissues were either frozen for analytical determinations, fixed in 80% ethanol for 72 h and included in paraffin for H&E staining, or fixed in paraformaldehyde (PFA) 4% and included in OCT (Thermo Scientific, Waltham, MA, USA) and stored in −80 °C for the evaluation of mHTT aggregates.

### 4.2. Preparation of 4QMn

Metal ligand Pytren-4Q was synthesized as reported previously [11]. In each test, 4QMn was prepared using equimolar solutions of pytren-4Q and MnSO4. Doses of 4QMn were prepared in 0.9% NaCl for IP delivery, in Tris-HCl 1M pH 7.2 IG or IN infusions, and sterile water for ad libitum administration.

### 4.3. Quantification of ALT Activity

The quantification of ALT activity in plasma was performed as indicated in the ALT Colorimetric Activity Assay kit (Cayman Chemicals, Ann Arbor, MI, USA).

### 4.4. Determination of Pytren-4Q in Tissues

Detection of Pytren-4Q was carried out by liquid chromatography UPLC-MS/MS in the Analytical Unit of the Health Research Institute Hospital La Fe (IIS La Fe, Valencia, Spain). Standard solutions (10–200 ng/mL) in water:acetonitrile (90:10, *v*/*v*) were prepared by diluting a stock solution of pytren-4Q 5 mg/mL in methanol. Frozen tissue samples of approximately 100 mg were placed in 2 mL tubes containing CK14 ceramic beads (Precellys, Saint Quentin en Yvelines, France) and 500 μL of ethyl acetate, and homogenized twice for 40 s at 6000 rpm at 4 °C in a Precellys 24 Dual system equipped with a Criolys cooler (Precellys). After that, they were centrifuged at 3000× *g* for 5 min at 4 °C. Supernatants were transferred to clean tubes and a second extraction was performed with 500 μL of ethyl acetate. Supernatants were pooled and evaporated to dryness in a Savant speedvac concentrator, reconstituted in 100 μL of water:acetonitrile (90:10, *v*/*v*), centrifuged at 10,000× *g* for 10 min at 4 °C, and transferred into 96-well plates for analysis. Analyses were performed in an Acquity UPLC system (Waters, Wilmslow, UK) equipped with an Acquity UPLC BEH C18 column (1.7 μm, 2.1 × 100 mm; Waters, Wilmslow, UK) with water and acetonitrile with 0.1% formic acid as mobile phase and a Waters Xevo TQ-S mass spectrometer (Waters, Wilmslow, UK) with an ESI source working in positive-ion mode and MRM mode for the MS analysis.

### 4.5. Immunohistochemistry Evaluation of Huntingtin Aggregates in Striatum

Striatum OCT blocks were cut into 10 μm slices, dried, and stored at −20 °C. After three washes with PBS 1× to remove the OCT, samples were permeabilized with Triton X-100 0.3% for 10 min, blocked for 1 h with Triton X-100 0.3% in 10% FBS, and incubated for 24 h with the primary antibody EM48 (MAB5374; Sigma, St. Louis, MO, USA) diluted 1:500 in the blocking solution. After three washes with PBS, the samples were incubated with the secondary antibody Alexa Fluor^®^ 488 (ab150113; Abcam, Cambridge, UK) diluted 1:500 in blocking solution and DAPI (ab228549; Abcam, Cambridge, UK). Finally, the samples were washed three times with PBS 1× and assembled with Fluorsave™ fluorescence preservation medium (Sigma, St. Louis, MO, USA).

Fluorescence images were obtained using an INCell Analyzer 2200 automated fluorescence microscope (Cytiva, Marlborough, MA, USA), equipped with a solid-state lighting source, different magnification targets, and fluorescence excitation/emission filters. Images were collected with a 16-bit sCMOS camera using the 20×/0.45NA lens, and two pairs of dichroic excitation/emission filters: 390/18 excitation and 432.5/48 emission for DAPI, and 475/28 excitation and 511.5/23 emission for the detection of antibody against huntingtin conjugated with FITC. The images were analyzed with the INCell Developer Toolbox program (Cytiva, Marlborough, MA, USA). The workflow for the analysis of the samples consisted of the segmentation of the nuclei based on the fluorescence of the DAPI, and the segmentation of the granules/accumulations of huntingtin based on the fluorescence of the FITC. After segmentation, a series of mathematical algorithms were applied to calculate the number of nuclei, the number and area of huntingtin accumulations, and the mHTT accumulations in the cell nuclei.

### 4.6. Quantification of Gene Expression

Gene expression was determined by reverse transcriptase polymerase chain reaction (RT-qPCR) using standard Molecular Biology Techniques and commercially available reagents and kits. RNA was extracted with TRIzol™ (Thermo Fisher, Waltham, MA, USA) in a TissueLyser LT cell disruptor (Qiagen, Hilden, Germany) and purified with the RNeasy^®^ Mini Kit (Qiagen, Hilden, Germany). Retrotranscription was performed with PrimeScript RT Reagent Kit (Takara, Paris, France) in a thermocycler UnoCycler 732–1200 (VWR, Darmstadt, Germany). For RT-qPCR, TB Green^®^ Premix Ex Taq™ kit (Takara, Paris, France) was used with primers (Darpp32 F TGC CTA TAC GCC CCC ATC AC and R CCC GAA GCT CCC CTA ACT CA), (Fan 1 F GCA CAC TTC TAC ATC AGC CCC and R GAG GTG AAA GGC CCC AGT GA) (Mlh1 F CAA GCA TCT CCT CGT CCT CCC and R CTT GAC GCC TTT CTG CAG CC), (Nfl F AAC AAG GTC CTG GAA GCC GA and R CCT GCT TCT CGT TAG TGG CG) and (Ubc F CCC AAG AAC AAG CAC AAG GAG G and R ACG TCG AGC CCA GTG TTA C), using a thermal cycler QuantStudio 5 Applied Biosystems (Thermo Fisher Scientific, Waltham, MA, USA) and a PCR program consisting of a denaturation step at 95 °C for 3 min and 40 cycles of 30 s at 94 °C, 07 s at 60 °C and 30 s at 72 °C, with a final extension of 3 min at 72 °C. The mRNA levels (2^−ΔCt^) of each gene were calculated by subtracting the Ct value for Ubc from the Ct value for Darpp32, Fan1, Mlh1or Nfl.

### 4.7. Statistical Analysis

Statistical analyses were conducted with GraphPad Prism software, version 8 (GraphPad, San Diego, CA, USA). Data are represented as mean ± SEM or otherwise specifically indicated. The ordinary one-way and two-way ANOVA tests and unpaired t-tests were applied for the analysis. Statistical significance was set at *p* < 0.05, 95% confidence.

## 5. Patents

4QMn compound is subjected to several patents: “Metallic complexes mimetic of SOD” (Spain 2355784B1, WO WO2011/033163, United States 9.145.386, Europe 2492270); “Use of SOD mimetic metal complexes as food agents and as cosmetic, patent addition” (Spain 2543850B1, WO WO2015/124824, United States US9.570.677); and “New European 4Q application and autophagy for neurodegenerative diseases” (Europe PCT-07877, WO PCT/EP2018/068010).

## Data Availability

Data will be available upon request.

## Supplementary Materials

The following supporting information can be downloaded at: https://www.mdpi.com/article/10.3390/ijms242015153/s1.
