# Supplementary material for: Small Molecule Pytren-4QMn Metal Complex Slows down Huntington’s Disease Progression in Male zQ175 Transgenic Mice
Source: Int J Mol Sci. 2023 Oct 13;24(20):15153. doi: 10.3390/ijms242015153 (PMC10607077; doi:10.3390/ijms242015153)
Supplement: Supplementary file 1 [file ijms-24-15153-s001.zip › ijms-2609158-supplementary.pdf]

Suppl. Table S1. Maximum tolerated doses

Compound	Dosage (mg/kg)	Mice C57BL/6J	Total observation time (h)	Death
IG	25	3 ♂ + 3 ♀	48	0
	200	3 ♂ + 3 ♀	48	0
	500	3 ♂ + 3 ♀	48	0
	2000	1 ♂	1	1
	1000	1 ♂	48	1
IP	1	3 ♂ + 3 ♀	48	0
	10	1 ♂	1	paralysis

Suppl. Table S2. Acute toxicity evaluation.

Compound	IP Dosage	Mice C57BL/6J	Total study time (d)	Death
NaCl	0,90%	2 ♂ + 3 ♀	14	0
4QMn	0,1 mg/kg	3 ♂ + 2 ♀	14	0
	1 mg/kg	2 ♂ + 3 ♀	14	0

Suppl. Table S3. Chronic toxicity evaluations.

Compound	Administration	IP Dosage	Mice C57BL/6J	Total study time (days)	Death
NaCl	IP	0,90%	3 ♂ + 2 ♀	28	0
4QMn		1 mg/kg	2 ♂ + 3 ♀	28	0
Tris-HCl	IG	1 M pH 7,2	1 ♂ + 2 ♀	90	1
4QMn		5 mg/kg	2 ♂ + 1 ♀	90	1
		10 mg/kg	1 ♂ + 2 ♀	90	0
		50 mg/kg	2 ♂ + 1 ♀	90	2

Suppl. Table S4. Detection of 4QMn in mouse brain and liver tissues.

Sample	Time (min)	Dose mg/kg	Administration via	Detected/Not detected	Area 391>207	Area 391>164
Brain 1	15	5	IN	Detected	3042	2563
Brain 2	15	5	IN	Detected	1197	1166
Brain 3	15	5	IN	Detected	1804	1402
Brain 4	15	50	IG	Detected	495	338
Brain 5	30	50	IG	Detected?	217	< LOD
Liver 4	15	50	IG	Detected	11098	8505
Liver 5	30	50	IG	Detected	2425	1945

Suppl. Table S5.Detection of 4QMn in blood serum after an IG infusion of 20 mg/kg.

Mg/kg.	Time (hours)	Area 391>207	Area 391>164
Blank		365	215
Standard 25 ppb		1040	1400
Standard 50 ppb		2744	4094
Standard 100 ppb		4359	6695
Serum 1	0.5	343	396
Serum 2	0.5	1965	2423
Serum 3	1	0	0
Serum 4	1	539	639
Serum 5	2	820	802
Serum 6	2	330	327

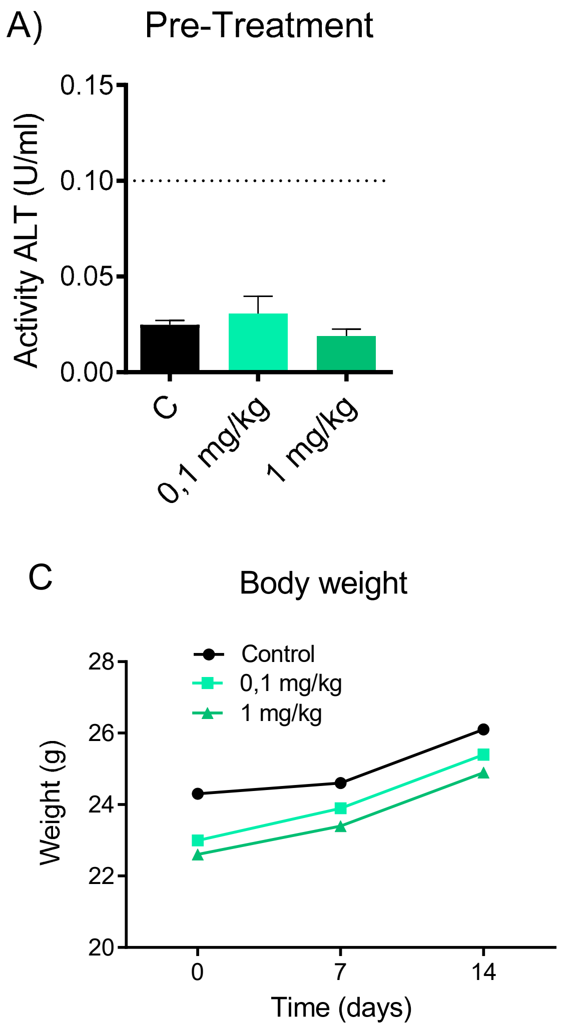

Suppl. Figure S1. Acute toxicity evaluation. (A-B) Plasma levels of ALT at days 0 and 14 after a single IP injection of NaCl or 4QMn. (C) Body weight measurements.

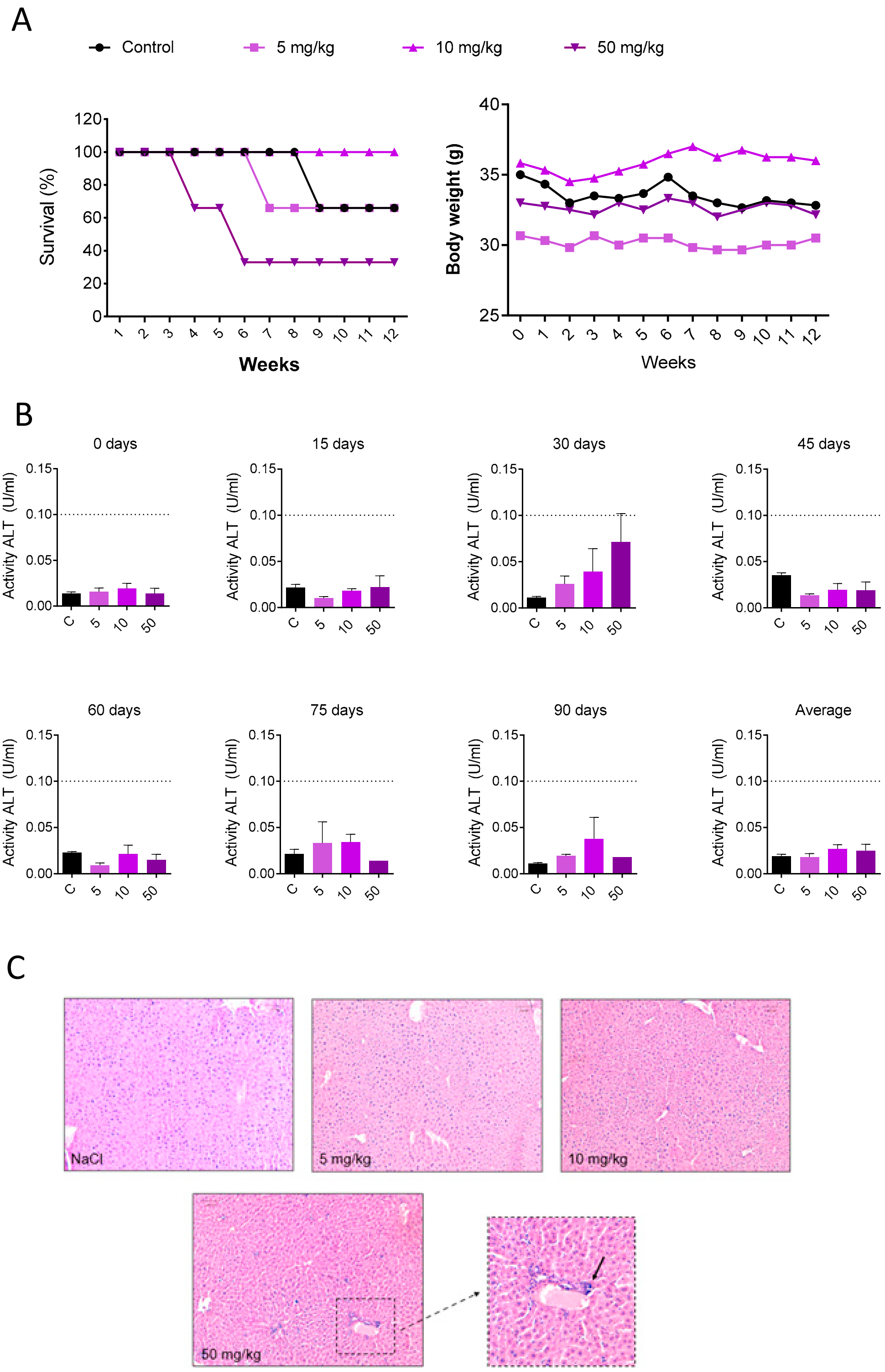

Suppl. Figure S2. Chronic IG toxicity evaluation. (A) Survival and body weight curves. (B) Plasma levels of ALT at the indicated times and average of all determinations (last panel). All data in were statistically analyzed using ordinary unidirectional ANOVA test comparing treated samples versus control (untreated) samples. Statistical significance was set at p<0.05,95% confidence. Graphical results were represented as mean ± SEM (standard error of mean) normalizing untreated group as a reference control. (C) Representative mages of H&E-stained liver section of mice administered IG with NaCl 0.9% or 4QMn

at 5, 10 or 50 mg/kg every three days for 90 days. Note the hepatic inflammation area at the 50 mg/kg dose. Magnification 10×.

A

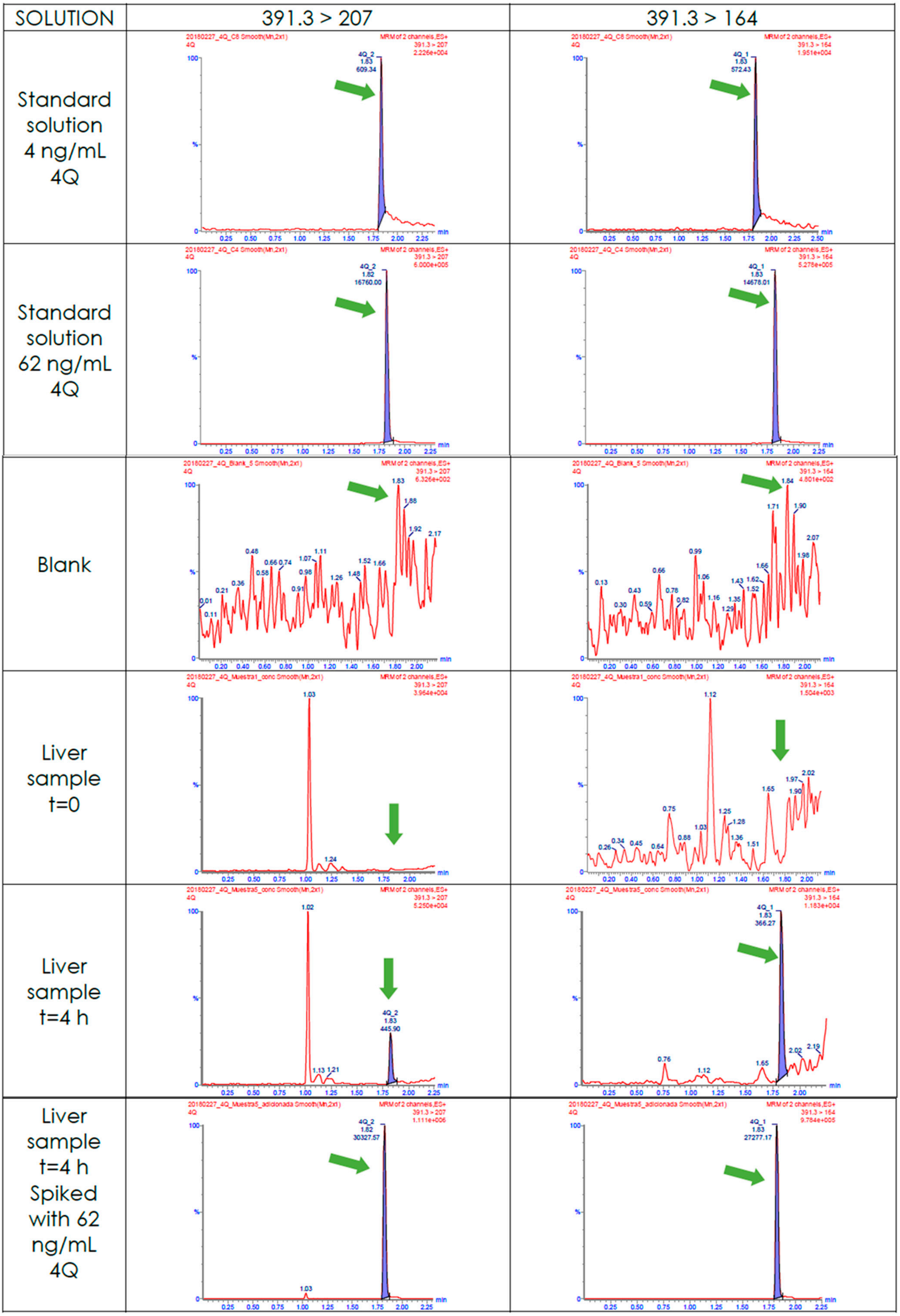
SOLUTION
391.3 > 207
391.3 > 164
Standard solution 4 ng/mL 4Q
Standard solution 62 ng/mL 4Q
Blank
Liver sample t=0
Liver sample t=4 h
Liver sample t=4 h Spiked with 62 ng/mL 4Q

B

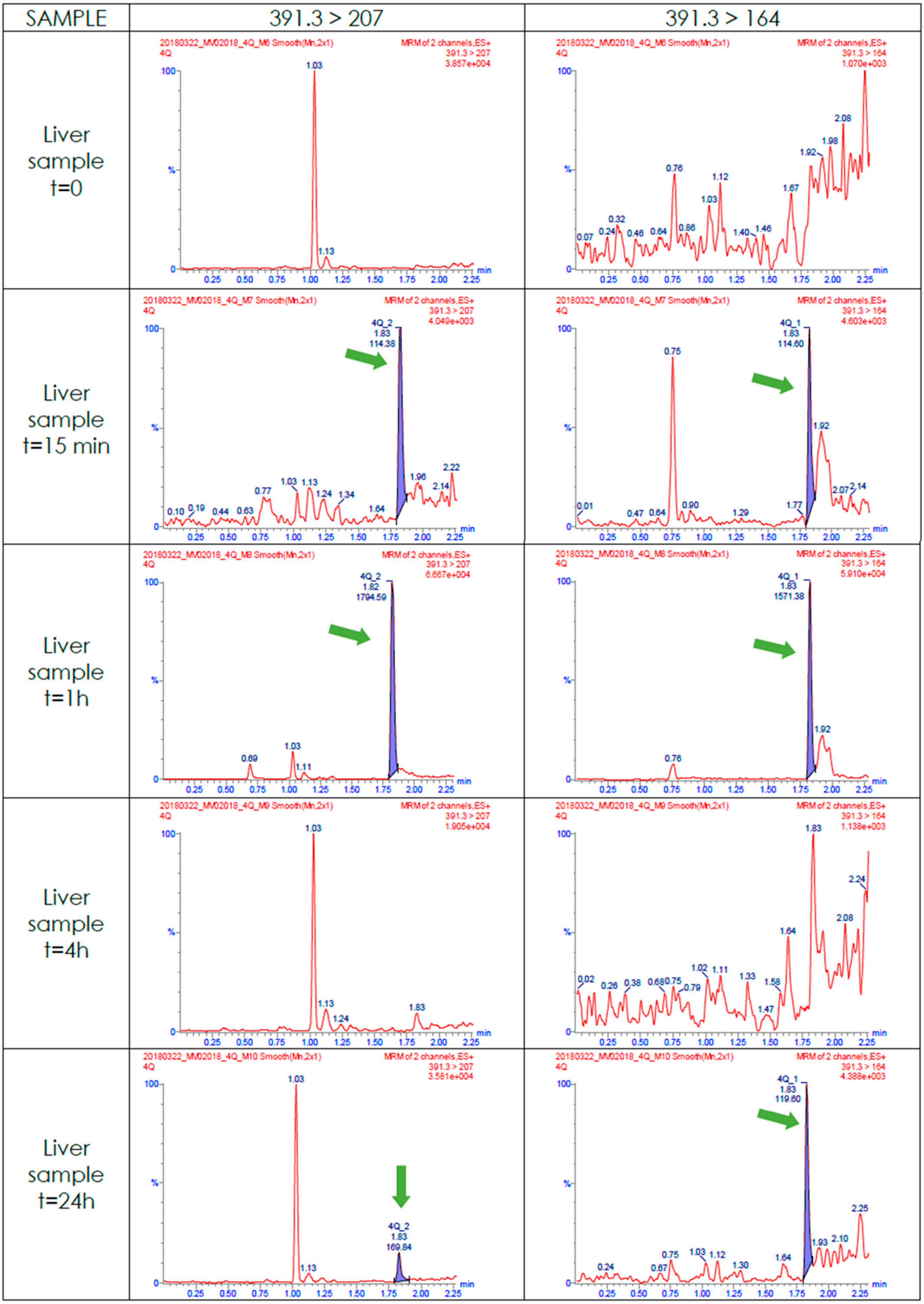
SAMPLE
391.3 > 207
391.3 > 164
Liver sample t=0
Liver sample t=15 min
Liver sample t=1h
Liver sample t=4h
Liver sample t=24h
20180322_MV02018_4Q_M6 Smooth(Mn,2x1)
20180322_MV02018_4Q_M7 Smooth(Mn,2x1)
20180322_MV02018_4Q_M8 Smooth(Mn,2x1)
20180322_MV02018_4Q_M9 Smooth(Mn,2x1)
20180322_MV02018_4Q_M10 Smooth(Mn,2x1)
4Q
MRM of 2 channels,ES+
391.3 > 207
391.3 > 164
3.857e+004
1.070e+003
4.049e+003
4.603e+003
6.667e+004
5.910e+004
1.905e+004
1.138e+003
3.581e+004
4.388e+003
4Q_2
1.83
114.38
4Q_1
1.83
114.60
4Q_2
1.82
1794.59
4Q_1
1.83
1571.38
4Q_2
1.83
169.84
4Q_1
1.83
119.60
100
%
0
min

C

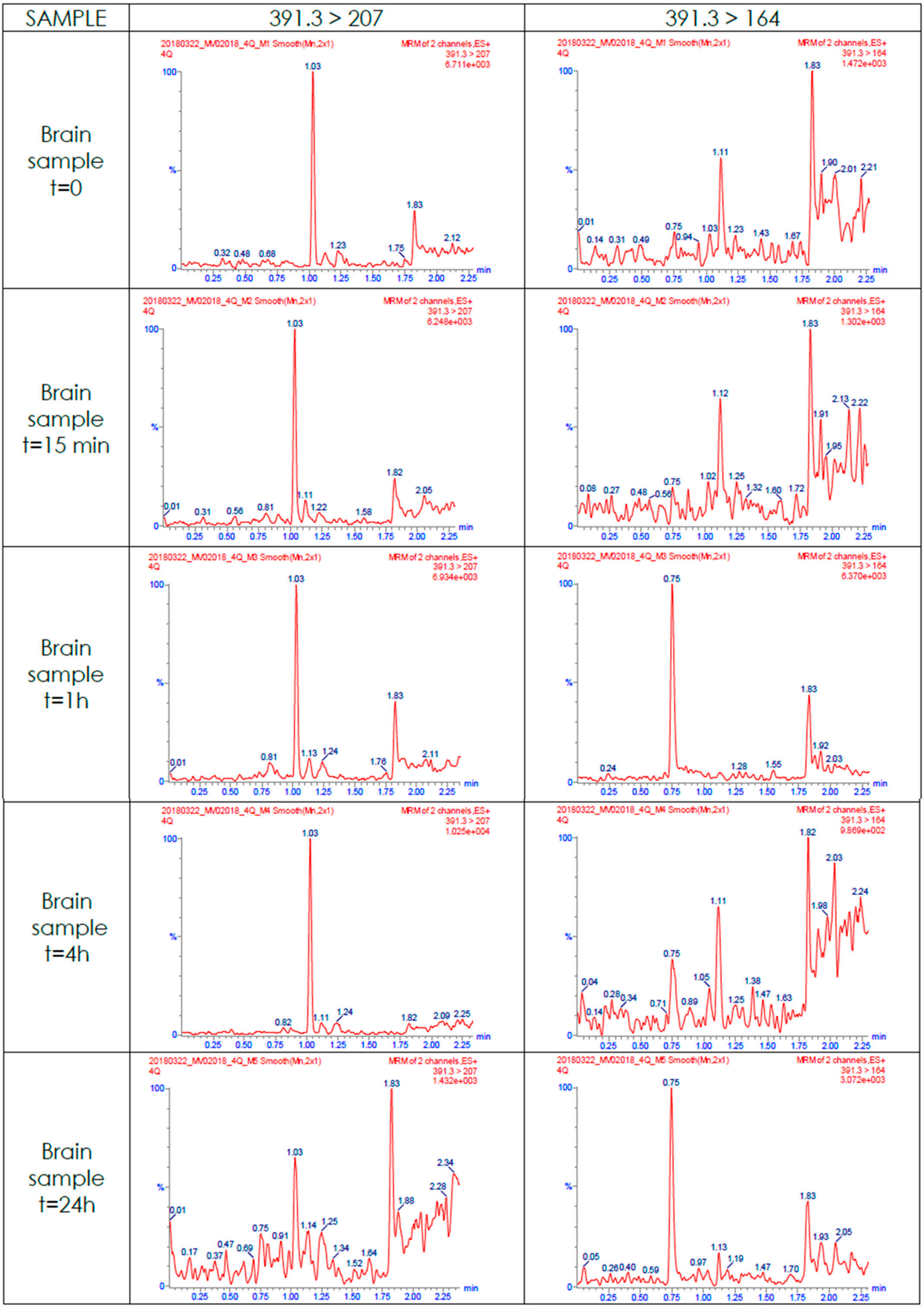
SAMPLE
391.3 > 207
391.3 > 164
Brain sample t=0
Brain sample t=15 min
Brain sample t=1h
Brain sample t=4h
Brain sample t=24h
20180322_MV02018_4Q_M1 Smooth(Mn,2x1)
20180322_MV02018_4Q_M2 Smooth(Mn,2x1)
20180322_MV02018_4Q_M3 Smooth(Mn,2x1)
20180322_MV02018_4Q_M4 Smooth(Mn,2x1)
20180322_MV02018_4Q_M5 Smooth(Mn,2x1)
MRM of 2 channels,ES+
6.711e+003
1.472e+003
6.248e+003
1.302e+003
6.934e+003
6.370e+003
1.025e+004
9.869e+002
1.432e+003
3.072e+003
min

Suppl. Figure S3. Detection of 4QMn in mouse liver. LC–MS–MS chromatograms of 4Q at two mass charge transitions: 391.3 > 207 and and 391.3 >164 after an IG infusion of 20 mg/Kg. (A-B) Detection in liver samples. (C) Corresponding chromatograms of brain samples.

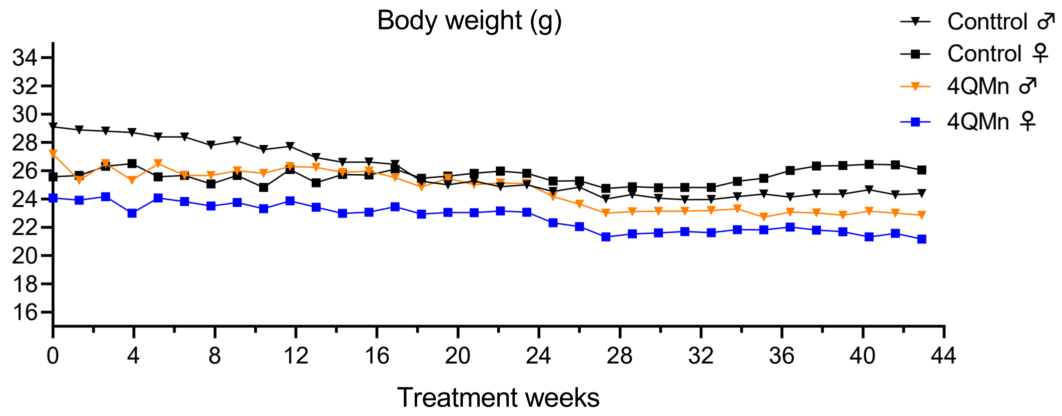

Suppl. Figure S4. Ten months body weight variation of 4QMn treated and control zQ175neo male and female mice. Mice were approximately 12 months of age at the star of the assay. The graph represents the average body weight of 5 control and 6 treated male mice and 6 control and 6 treated female mice.
